# Supplementary figures and images for: Refractory inflammatory arthritis definition and model generated through patient and multi-disciplinary professional modified Delphi process
Source: PLoS One. 2023 Aug 9;18(8):e0289760. doi: 10.1371/journal.pone.0289760 (PMC10411820; doi:10.1371/journal.pone.0289760)

**Supplementary Figure S6: Overview of Definition Development and Refinement**

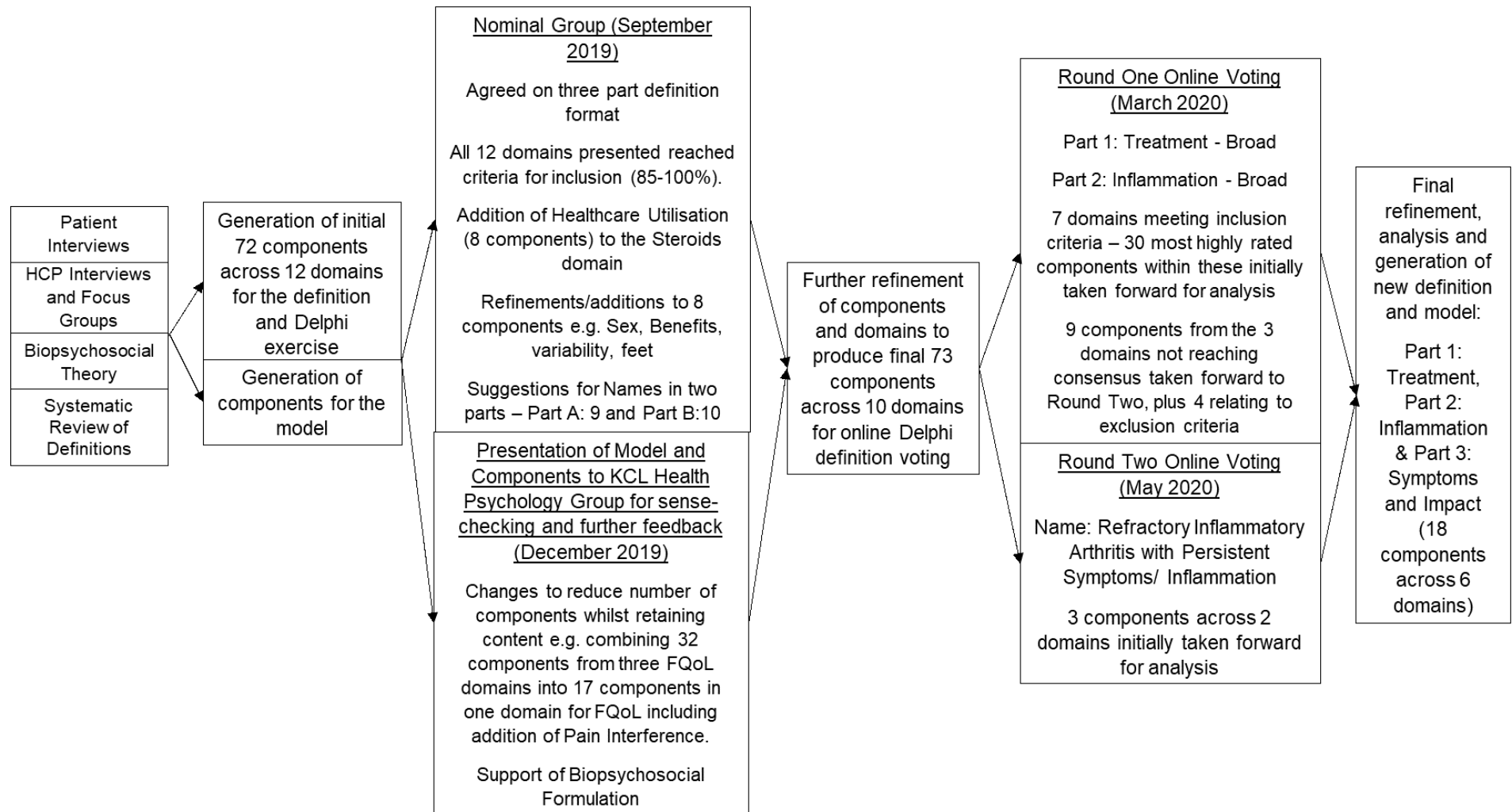

Supplement: S3 Fig — (PDF) [file pone.0289760.s003.pdf]
