# Supplementary material for: Refractory inflammatory arthritis definition and model generated through patient and multi-disciplinary professional modified Delphi process
Source: PLoS One. 2023 Aug 9;18(8):e0289760. doi: 10.1371/journal.pone.0289760 (PMC10411820; doi:10.1371/journal.pone.0289760)
Supplement: S4 Fig — (PDF) [file pone.0289760.s004.pdf]

Supplementary Figure S8: Round One Rankings of Domains by Role Group

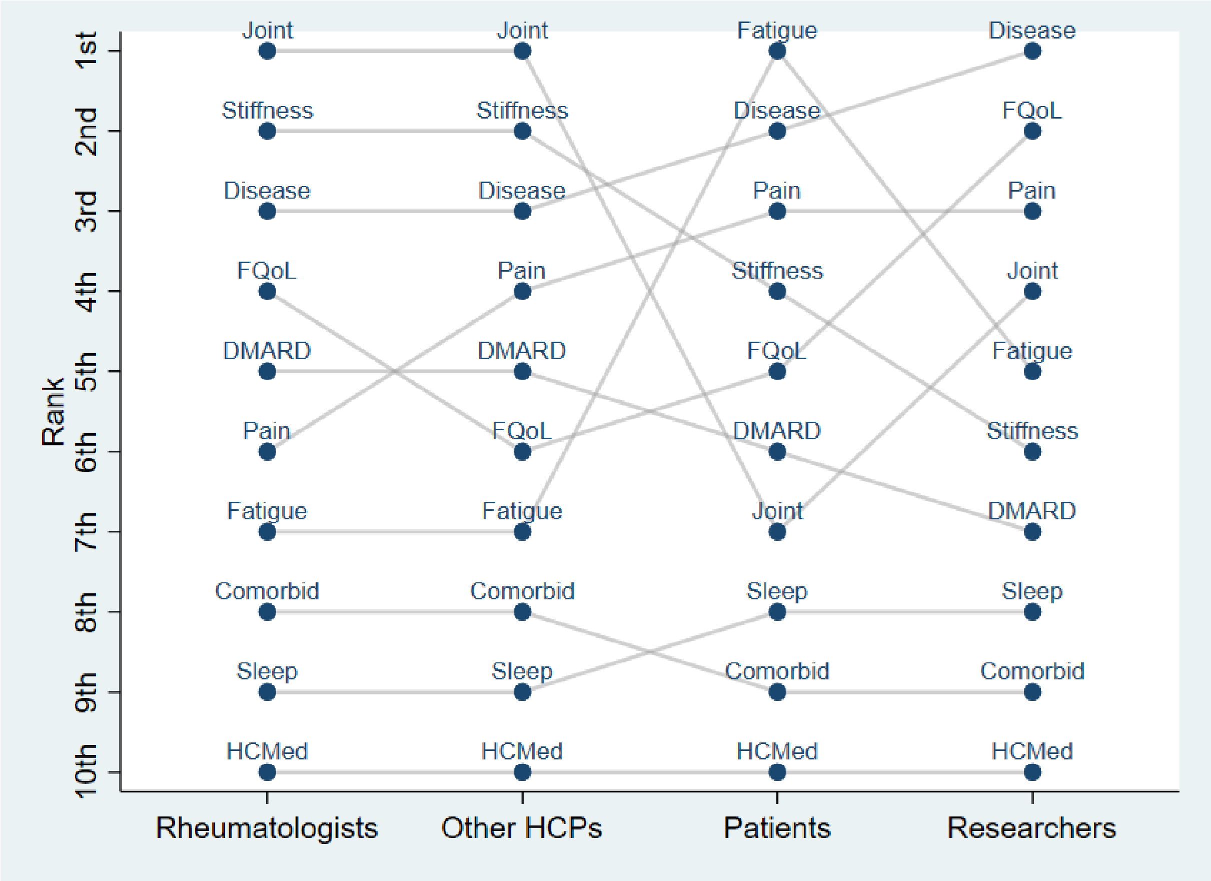

Please note: Joint = Joint Activity, Disease = Disease Activity, FQoL = Functioning and Quality of Life, DMARD = DMARD Experiences, Comorbid = Comorbidities and Wider Involvement Outside of Joints, HCMed = Healthcare and Medications Utilisation
