# Supplementary material for: Refractory inflammatory arthritis definition and model generated through patient and multi-disciplinary professional modified Delphi process
Source: PLoS One. 2023 Aug 9;18(8):e0289760. doi: 10.1371/journal.pone.0289760 (PMC10411820; doi:10.1371/journal.pone.0289760)
Supplement: S2 Table — (PDF) [file pone.0289760.s007.pdf]

Supplementary Table S11: Assessments initially mapped onto Refractory Inflammatory Arthritis definition

RIA Definition		Assessment Methods (including cut-offs)
Part 1: Treatment		Treatment History (≥1 csDMARDs, and ≥1 anti-TNF/b/tsDMARDs) (Watts et al., 2013)
Part 2: Inflammation		Abnormal/Raised Inflammatory markers (ESR / CRP based on the local laboratory standards) (Watts et al., 2013)
		Physical examination (TJC/SJC) (Watts et al., 2013)
		Imaging (Any) (Watts et al., 2013)
Part 3: Symptoms and Impact	1. Disease Activity	Clinical Notes / Consultation / Medical and Treatment History (Watts et al., 2013) DAS28 >3.2 (van der Heijde et al., 1990) or SDAI >11 (Smolen et al., 2003)
		RAID (scoring higher on Q5) (Gossec et al., 2011)
	2. Joint Involvement	(C)HAQ (Symptoms Section) (Fries et al., 1980; Singh et al., 1994)
		MSK-HQ (scoring 0-2 on Q1 and/or 2) (Hill et al., 2016)
		Consultation / Joint Examination / Clinical Notes (Watts et al., 2013)
	3. Pain	MSK-HQ (scoring 0-2 on Q1 and/or 2) (Hill et al., 2016)
		VAS >3.5 (Boonstra et al., 2014)
		EQ5D (scoring 2 or 3 on Q4) (EuroQol Group, 1990)
		RAID (scoring higher on Q1) (Gossec et al., 2011)
	4. Fatigue	BRAF (Higher total score and on items) (Nicklin et al., 2010)
		VAS >2.0 (Pollard et al., 2006)
		General Fatigue MSK-HQ (scoring 0-2 on Q10) (Hill et al., 2016)
		RAID (scoring higher on Q3) (Gossec et al., 2011)

Please note: DAS28: Disease Activity Score 28 joint count, SDAI: Simplified Disease Activity Index, RAID: Rheumatoid Arthritis Impact of Disease, (C)HAQ: Child or Adult Health Assessment Questionnaire, MSK-HQ: Musculoskeletal Health Questionnaire, VAS: Visual Analogue Scale, EQ5D: EuroQol 5-Dimensions, BRAF: Bristol RA Fatigue Scale

	RIA Definition	Assessment Methods (including cut-offs)
Part 3: Symptoms and Impact	5. Functioning and Quality of Life	(C)HAQ ≥1.5 (Fries et al., 1980; Singh et al., 1994)<br>EQ5D (scoring 2 or 3 on Q1-3 and Q5) (EuroQol Group, 1990)<br>WSAS (higher total score and on items 1-4) (Mundt et al., 2002)<br>MSK-HQ (scoring 0-2 on Q3-7 and Q11) (Hill et al., 2016)<br>RAID (scoring higher on Q2 and Q6) (Gossec et al., 2011)<br>RADS (Silke et al., 2021)<br>Consultation (Watts et al., 2013)
	6. cs/b/tsDMARD Experiences	Clinical Notes / Treatment and DAS28 History / Consultation (Watts et al., 2013)

Please note: (C)HAQ: Child or Adult Health Assessment Questionnaire, MSK-HQ: Musculoskeletal Health Questionnaire, EQ5D: EuroQol 5-Dimensions, Work and Social Adjustment Scale, RADS: Rheumatoid Arthritis Distress Scale.
