# Supplementary material for: Refractory inflammatory arthritis definition and model generated through patient and multi-disciplinary professional modified Delphi process
Source: PLoS One. 2023 Aug 9;18(8):e0289760. doi: 10.1371/journal.pone.0289760 (PMC10411820; doi:10.1371/journal.pone.0289760)
Supplement: S3 Table — A) Data capture and synthesis of measures used in the RIA definition across RA/JIA Biologic registries/cohort (Location, Condition and Parts One and Two). B) Data capture and synthesis of measures used in the RIA definition across RA/JIA Biologic registries/cohort (Part Three). (PDF) [file pone.0289760.s008.pdf]

Supplementary Tables S12: A) Data capture and synthesis of measures used in the RIA definition across RA/JIA Biologic registries/cohort (Location, Condition and Parts One and Two)

Name and Reference of Registry or Cohort	Location	Condition	Part One	Part Two		
			Treatment History	ESR or CRP	T/SJC or A/LJC	Imaging (US or MRI)
ABC (Otten et al., 2013)	Netherlands	JIA	✓	✓	✓	✓
ABioPharm (Barnabe et al., 2018)	Canada	RA	×	✓	✓	×
ACTION (Nüßlein et al., 2014)	Worldwide	RA	✓	✓	✓	×
ATTRA (Horák, Skácelová, Hejduk, Smržová, & Pavelka, 2013)	Czech Republic	RA	✓	✓	✓	×
ARAD (Williams, Buchbinder, March, & Lassere, 2011)	Australia	RA & JIA	✓	✓	✓	×
ARAMIS (Singh, 2001)	USA and Canada	RA	✓	✓	✓	×
ARRRA (Karateev et al., 2015)	Russia	RA & JIA	✓	✓	✓	×
ARTIS (Askling et al., 2006)	Sweden	RA	✓	✓	✓	×
BARFOT (Hafström et al., 2019)	Sweden	RA	✓	✓	✓	×
BCRD and BSPAR-ETN (Kearsley-Fleet et al., 2016) (Hyrich, 2022)*	UK	JIA	✓	✓	✓	×
BIKER (Horneff et al., 2009; Klotsche et al., 2014)	Germany and Austria	RA	✓	✓	✓	×
BIOBADASER (Sanchez-Piedra et al., 2019)	Spain	RA and JIA	✓	✓	✓	×
BIOCURA (Cuppen et al., 2016) (Hiligsmann, Rademacher, Kaal, Bansback, & Harrison, 2018)*	Netherlands	RA	✓	✓	✓	×
BIOREG (Rintelen et al., 2016)	Austria	RA	✓	✓	✓	×
BioRx.si (Rotar, Hočevar, Rebolj Kodre, Praprotnik, & Tomšič, 2015)	Slovenia	RA	✓	✓	✓	×
BioTRAC (Thorne et al., 2014)	Canada	RA	✓	✓	✓	×
BRASS (Iannaccone et al., 2010)	UK	RA	✓	✓	✓	×
BSR-BR (Hyrich, 2018)	UK	RA	✓	✓	✓	×
CAPS (Adib et al., 2008; Hanns et al., 2016; McErlane et al., 2013)	UK	JIA	✓	✓	✓	×
CARRA (BeukelmanKimura, et al., 2017)	USA and Canada	JIA	✓	✓	✓	✓
CATCH (Bykerk et al., 2012)	Canada	RA	✓	✓	✓	×
CONAART (Marcos et al., 2010) (Hiligsmann et al., 2018)*	Argentina	RA	✓	✓	✓	×
CORRONA (Kremer, 2016)	USA	RA	✓	✓	✓	×
DANBIO (Ibfelt, Jensen, & Hetland, 2016)	Denmark	RA	✓	✓	✓	×
DREAM (de Punder et al., 2012) (Hiligsmann et al., 2018)*	Netherlands	RA	✓	✓	✓	×
ERAS and ERAN, (Young et al., 2011)	UK	RA	Mainly csDMARD	✓	✓	×
ESPOIR (Combe et al., 2007)	France	RA	✓	✓	✓	✓

Please note: ESR = Erythrocyte Sedimentation Rate, CRP = C-Reactive Protein, T/SJC = Tender/Swollen Joint Count, A/LJC = Active/Limited Joint Count, ABC = Arthritis and Biologicals in Children, ABioPharm = Alberta Biologics Pharmacosurveillance Program, ACTION = Abatacept In Routine Clinical Practice, ATTRA = Czech Biologics Registry, ARAD = Australian Rheumatology Association Database, ARAMIS = Arthritis, Rheumatism and Aging Medical Information System, ARRRA = All-Russian Register of patients with Rheumatoid Arthritis, ARTIS = Swedish National Biologics Registry, BARFOT = Better Anti-Rheumatic Pharmacotherapy, BCRD = Biologics for Children with Rheumatic, Diseases, BSPAR-ETN = British Society for Paediatric and Adolescent Rheumatology – Etanercept, BIKER = German Biologics JIA Registry, BIOBADASER = Spanish registry of adverse events involving biological therapies in rheumatic diseases, BIOCURA = Biologicals and Outcome Compared and Predicted in Utrecht Region in Rheumatoid Arthritis study, BIOREG = Austrian Biologics Registry for inflammatory rheumatic disease, BioRx.si, = Slovenian prospective national on-line biologics registry, BioTRAC = Biologic Treatment Registry Across Canada, BRASS = Brigham and Women's Hospital Rheumatoid Arthritis Sequential Study, BSR-BR = British Society of Rheumatology Biologics Register, CAPS = Childhood Arthritis Prospective Study, CARRA = Childhood Arthritis and Rheumatology Research Alliance, CATCH = Canadian Early Arthritis Cohort, CONAART = Argentine Consortium for Early Arthritis, CORRONA = Consortium of Rheumatology Researchers of North America, DANBIO = Danish biologics register for rheumatologic diseases, DREAM = Dutch Rheumatoid Arthritis Monitoring registry, ERAS/N = Early Rheumatoid Arthritis Study/Network, ESPOIR = Etude et Suivi des Polyarthrites Indifférenciées Récentes.

Name and Reference of Registry or Cohort	Location	Condition	Part One	Part Two		
			Treatment History	ESR or CRP	T/SJC or A/LJC	Imaging (US or MRI)
GISEA (Lapadula, Ferraccioli, Ferri, Punzi, & Trotta, 2011)	Italy	RA	✓	✓	✓	Not clear
GLADAR (Massardo et al., 2012)	Latin America	RA	×	✓	✓	×
ICON (Sengler et al., 2015)	Germany	JIA	✓	✓	✓	×
IMPARTS (Matcham, Norton, Steer, & Hotopf, 2016; Rayner et al., 2014)	UK	RA	×	✓	✓	×
IORRA (Yamanaka et al., 2007) (Hiligsmann et al., 2018)*	Japan	RA	✓	✓	✓	×
JACS (Beukelman, Anink, et al., 2017)	Australia	JIA	Not Clear	✓	✓	×
JUMBO (Minden et al., 2012)	Germany	JIA	✓	✓	✓	×
METEOR (van den Berg, van der Heijde, Landewé, van Lambalgen, & Huizinga, 2014)	Worldwide	RA	✓	✓	✓	×
NDB (Zink, Listing, Klindworth, & Zeidler, 2001) (Hiligsmann et al., 2018)*	Germany	RA	✓	✓	✓	×
NDB (Wolfe & Michaud, 2011)	USA	RA	✓	✓	✓	×
NEIAA (British Society of Rheumatology, 2019)	UK	RA & JIA	Some	✓	✓	×
Nijmegen Early RA Cohort (Welsing & van Riel, 2004) (Hiligsmann et al., 2018)*	Netherlands	RA	✓	✓	✓	×
NoAR (Druce, Jones, Macfarlane, Verstappen, & Basu, 2015; Symmons & Silman, 2003)	UK	RA	Mainly csDMARD	✓	✓	×
Nordic JIA Cohort (Nordal et al., 2011)	Sweden, Finland, Denmark, Norway, Iceland	JIA	✓	✓	✓	×
NoRDMARD (Olsen, Haavardsholm, Moholt, Kvien, & Lie, 2014)	Norway	RA & JIA	✓	✓	✓	×
NPRD (Minden, Niewerth, Listing, & Zink, 2002)	Germany	JIA	Mainly csDMARD	Not clear	✓	×
ORAR (Kvien & Uhlig, 2004)	Norway	RA	✓	✓	✓	×
PHARMACHILD (Beukelman, Anink, et al., 2017)	Worldwide	JIA	Not Clear	✓	✓	Not clear
QUEST-RA (Sokka et al., 2007)	Worldwide	RA	✓	✓	✓	×
RABBIT (Gerhold et al., 2015)	Germany	RA	✓	✓	✓	×
RADIUS1 (Gibofsky et al., 2011)	USA	RA	✓	✓	✓	×
ReACCh Out (Oen et al., 2009)	Canada	JIA	✓	✓	✓	×
REACH (Alves et al., 2011; Kuijper et al., 2014)	Netherlands	RA	✓	✓	✓	×
REUMA.PT (Canhão, Faustino, Martins, & Fonseca, 2011)	Portugal	RA & JIA	✓	✓	✓	×
Rhumadata© (Choquette et al., 2019)	Canada	RA	✓	✓	✓	×

Please note: ESR = Erythrocyte Sedimentation Rate, CRP = C-Reactive Protein, T/SJC = Tender/Swollen Joint Count, A/LJC = Active/Limited Joint Count, GISEA = Italian Group for the Study of Early Arthritis, GLADAR = Grupo Latino Americano de Estudio de Artritis Reumatoide, ICON = Inception Cohort of Newly diagNosed patients with JIA,, IMPARTS = Integrating Mental & Physical healthcare: Research, Training & Services, IORRA = Institute of Rheumatology, Rheumatoid Arthritis, JACS = Juvenile arthritis cohort study, JUMBO = Juvenile arthritis MTX/Biologics long-term Observation, METEOR = Measurement of Efficacy of Treatment in the “Era of Outcome” in Rheumatology, NDB = national database of the German Collaborative Arthritis Centres, NDB = National Data Bank for Rheumatic Diseases, NEIAA = National Early Inflammatory Arthritis Audit, NoAR = Norfolk Arthritis Register, NoRDDMARD = Norwegian Antirheumatic Drug Register, NPRD = National Paediatric Rheumatology Database, ORAR = Oslo Rheumatoid Arthritis Register, PHARMACHILD = Pharmacovigilance in JIA patients treated with biologic agents and/or MTX, QUEST-RA = Quantitative Patient Questionnaires in Standard Monitoring of Patients with Rheumatoid Arthritis, RABBIT = German register for long-term observation of biologics in RA, RADIUS1 = Rheumatoid Arthritis Disease-Modifying Antirheumatic Drug Intervention and Utilization Study Cohort1, ReACCh Out = Research in Arthritis in Canadian Children emphasizing Outcomes, REACH = Rotterdam Early Arthritis Cohort, REUMA.PT = Rheumatic Diseases Portuguese Register.

Name and Reference of Registry or Cohort	Location	Condition	Part One	Part Two		
			Treatment History	ESR or CRP	T/SJC or A/LJC	Imaging (US or MRI)
ROB-FIN (Nordström et al., 2006)	Finland	RA	✓	✓	✓	×
RRBR (Codreanu, Mogosan, Ionescu, Ancuta, & Opris, 2014)	Romania	RA	✓	✓	✓	✓
SCQM (Uitz, Fransen, Langenegger, & Stucki, 2000)	Switzerland	RA	✓	✓	✓	×
SRQ (Eriksson, Askling, & Arkema, 2014)<br>(Hiligsmann et al., 2018)*	Sweden	RA	✓	✓	✓	×
STURE (Askling et al., 2006)	Sweden	RA	✓	✓	✓	×
Swedish JIA Register (Beukelman, Anink, et al., 2017)	Sweden	JIA	Not Clear	✓	✓	×
TURKBIO (Önen et al., 2022)	Turkey	RA	✓	✓	✓	×
VARAR (Mikuls et al., 2010)	USA	RA	✓	✓	✓	×
WC (Kovalchik, Charles-Schoeman, Khanna, & Paulus, 2012)	USA and Mexico	RA	Mainly csDMARD	✓	✓	×

Please note: ESR = Erythrocyte Sedimentation Rate, CRP = C-Reactive Protein, T/SJC = Tender/Swollen Joint Count, A/LJC = Active/Limited Joint Count, ROB-FIN = National Register of Biological Treatment in Finland, RRBR = Romanian Registry of Rheumatic Diseases, SCQM = Swiss Clinical Quality Management in Rheumatic Diseases, SRQ = Swedish Rheumatology Quality Registries, STURE = Stockholm TNF follow-Up Registry, TURKBIO = Turkish Biologics Register, VARAR = Veterans Affairs Rheumatoid Arthritis Registry, WC = Western Consortium of Practicing Rheumatologists.

Supplementary Tables S12: B) Data capture and synthesis of measures used in the RIA definition across RA/JIA Biologic registries/cohort (Part Three)

	Part Three																	
Name and Reference of Registry or Cohort	Treatment History	Clinical Notes	DAS28/JADAS or C/SDAI	RAID	Manifestations/ Features	Steroid Use	Stiffness	T/SJC or A/LJC	Imaging (Radiographs)	MSK-HQ	Pain VAS	EQ-5D	BRAF	Fatigue VAS	(C)HAQ	WSAS	RADS	Treatment Change Reasons
ABC (Otten et al., 2013)	✓	✓	✗	✗	✗	✓	✗	✓	✗	✗	✗ (PtVAS)	✗	✗	✗ (PtVAS)	✓	✗	✗	✓
ABioPharm (Barnabe et al., 2018)	✗	✗	✓	✗	✗	✗	✓	✓	✗	✗	✓	✓	✗ (SF-36)	✓	✓	✗ (study-specific)	✗ (SF-36)	✗
ACTION (Nüßlein et al., 2014)	✓	✓	✓	✗	✗	✓	✗	✓	✓	✗	✓	✗	✗	✗ (PtVAS)	✓	✗	✗	✓
ATTRA (Horák et al., 2013)	✓	✓	✓	✗	✗	✓	✗	✓	✗	✗ (SF-36)	✗ (PtVAS)	✓	✗ (SF-36)	✗ (SF-36)	✓	✗ (SF-36)	✗ (SF-36)	✓
ARAD (Williams et al., 2011)	✓	✓	✓	✓	✗	✓	✗	✓	✗	✗ (AQoL & PEDs-QL)	✗ (PtVAS)	✓	✗ (SF-36)	✗ (PtVAS)	✓	✗ (AQoL and PEDs-QL)	✗ (SF-36)	✓
ARAMIS (Singh, 2001)	✓	✓	✓	✗	✗	✓	✗	✓	✗	✗	✓	✗	✗ (SF-36)	✗ (SF-36)	✓	✗ (SF-36)	✗ (AIMS-DA & SF-36)	✓
ARRRA (Karateev et al., 2015)	✓	✓	✓	✗ (RAPID3)	✗	✓	✗	✓	✗	✗ (RAPID 3)	✗ (RAPID 3 & PtVAS)	✓	✗	✗ (PtVAS)	✓	✗	✗	✗
ARTIS (Askling et al., 2006)	✓	✓	✓	✗	✗	✓	✗	✓	✗	✗	✓	✗	✗	✗ (PtVAS)	✓	✗	✗	Not clear

Please note: T/SJC = Tender/Swollen Joint Count, A/LJC = Active/Limited Joint Count, DAS28 = Disease Activity Score-28 Joint count, JADAS = Juvenile Arthritis Disease Activity Score, C/SDAI = Clinical/Simplified Disease Activity Index, RAID = Rheumatoid Arthritis Impact of Disease, (C)HAQ = (Child) Health Assessment Questionnaire , MSK-HQ = Musculoskeletal Health Questionnaire, VAS = Visual Analogue Scale, EQ-5D = EuroQol 5 Dimensions, BRAF = Bristol Rheumatoid Arthritis Fatigue, WSAS = Work and Social Adjustment Scale, RADS = Rheumatoid Arthritis Distress Scale, ABC = Arthritis and Biologicals in Children, ABioPharm = Alberta Biologics Pharmacosurveillance Program, ACTION = Abatacept In Routine Clinical Practice, ATTRA = Czech Biologics Registry, ARAD = Australian Rheumatology Association Database, ARAMIS = Arthritis, Rheumatism and Aging Medical Information System, ARRRA = All-Russian Register of patients with Rheumatoid Arthritis, ARTIS = Swedish National Biologics Registry, RAPID3 = Routine Assessment of Patient Index Data 3, PtVAS = Patient Global VAS, SF-36 = Short Form – 36 Item, AQoL = Assessment of Quality of Life, Peds-QL = Pediatric Quality of Life Inventory, AIMS-DA = Arthritis Impact Measurement Scales depression and anxiety scales

Part Three																		
Name and Reference of Registry or Cohort	Treatment History	Clinical Notes	DAS28/JADAS or C/SDAI	RAID	Manifestations/ Features	Steroid Use	Stiffness	T/SJC or A/LJC	Imaging (Radiographs)	MSK-HQ	Pain VAS	EQ-5D	BRAF	Fatigue VAS	(C)HAQ	WSAS	RADS	Treatment Change Reasons
BARFOT (Hafström et al., 2019)	✓	✓	✓	×	×	✓	×	✓	✓	×	✓	×	×	× (PtVAS)	✓	×	×	Not clear
BCRD and BSPAR-ETN (Kearsley-Fleet et al., 2016) (Hyrich, 2022)*	✓	✓	✓	× (CHU -9D)*	×	✓	×	✓	×	× (CHU-9D)*	✓	× (EQ-5D-Y)*	×	× (PtVAS & CHU-9D)*	✓	× (CHU-9D)*	× (CHU-9D)*	✓
BIKER (Horneff et al., 2009; Klotsche et al., 2014)	✓	✓	× (PedACR)	× (Ped sQL)	×	✓	✓	✓	×	× (PedsQL)	× (PtVAS)	×	×	× (PtVAS)	✓	× (Peds QL)	× (Peds QL)	✓
BIOBADASER (Sanchez-Piedra et al., 2019)	✓	✓	✓	×	×	Not clear	×	✓	×	×	× (PtVAS)	×	×	× (PtVAS)	×	×	×	✓
BIOCURA (Cuppen et al., 2016) (Hiligsmann et al., 2018)*	✓	✓	✓	×	×	✓	×	✓	×	×	✓	✓	× (SF-36)*	× (SF-36)*	✓	× (SF-36)*	× (SF-36)*	Some
BIOREG (Rintelen et al., 2016)	✓	✓	✓	×	×	✓	×	✓	×	×	× (RADAI -5)	×	×	× (PtVAS)	✓	×	×	✓
BioRx.si (Rotar et al., 2015)	✓	✓	✓	×	×	✓	×	✓	×	×	✓	×	×	× (PtVAS)	✓	×	×	×
BioTRAC (Thorne et al., 2014)	✓	✓	✓	×	×	×	✓	✓	×	×	✓	×	×	× (PtVAS)	✓	×	×	✓

Please Note: T/SJC = Tender/Swollen Joint Count, A/LJC = Active/Limited Joint Count, DAS28 = Disease Activity Score-28 Joint count, JADAS = Juvenile Arthritis Disease Activity Score, C/SDAI = Clinical/Simplified Disease Activity Index,, RAID = Rheumatoid Arthritis Impact of Disease, (C)HAQ = (Child) Health Assessment Questionnaire , MSK-HQ = Musculoskeletal Health Questionnaire, VAS = Visual Analogue Scale, EQ-5D = EuroQol 5 Dimensions, BRAF = Bristol Rheumatoid Arthritis Fatigue, WSAS = Work and Social Adjustment Scale, RADS = Rheumatoid Arthritis Distress Scale, BARFOT = Better Anti-Rheumatic Pharmacotherapy, BCRD = Biologics for Children with Rheumatic, Diseases, BSPAR-ETN = British Society for Paediatric and Adolescent Rheumatology – Etanercept, BIKER = German Biologics JIA Registry, BIOBADASER = Spanish registry of adverse events involving biological therapies in rheumatic diseases, BIOCURA = Biologicals and Outcome Compared and Predicted in Utrecht Region in Rheumatoid Arthritis study, BIOREG = Austrian Biologics Registry for inflammatory rheumatic disease, BioRx.si, = Slovenian prospective national on-line biologics registry, BioTRAC = Biologic Treatment Registry Across Canada, PtVAS = Patient Global VAS, CHU-9D = Child Health Utility Index – 9 item, EQ-5D-Y = EuroQol 5 Dimensions Youth, PedACR = ACR paediatric response measure, Peds-QL = Pediatric Quality of Life Inventory, SF-36 = Short Form – 36 Item, RADAI(5) = Rheumatoid Arthritis Disease Activity Index (5 item)
*Collected but data Not currently published

Name and Reference of Registry or Cohort	Part Three																	
	Treatment History	Clinical Notes	DAS28/JADAS or C/SDAI	RAID	Manifestations/ Features	Steroid Use	Stiffness	T/SJC or A/LJC	Imaging (Radiographs)	MSK-HQ	Pain VAS	EQ-5D	BRAF	Fatigue VAS	(C)HAQ	WSAS	RADS	Treatment Change Reasons
BRASS (Iannaccone et al., 2010)	✓	✓	✓	✗	✓	✓	✓	✓	✗	✗ (ASES & SF-12)	✓	✓	✗	Not clear	✓	✗ (Social Support)	✗ (SF-12, MHI & PHQ-9)	✓
BSR-BR (Hyrich, 2018)	✓	✓	✓	✗	✓	✓	✗	✓	✗	✗ (SF-36 & B-IPQ)	✗ (PtVAS)	✓	✗ (SF-36)	✗ (SF-36)	✓	✗ (WPS)	✗ (SF-36 & B-IPQ)	✓
CAPS (Adib et al., 2008; Hanns et al., 2016; McErlane et al., 2013)	✓	✓	✓	✗	✓	✓	✗	✓	✗	✗	✓	✗	✗	✗ (PtVAS)	✓	✗	✗ (MFQ)	✗
CARRA (BeukelmanKimura, et al., 2017)	✓	✓	✓	✗ (PGH-7)	✓	✓	✓	✓	✓	✗ (PGH-7)	✓	✗	✗	✗ (PtVAS)	✓	✗ (PGH-7)	✗ (PGH-7)	✓
CATCH (Bykerk et al., 2012)	✓	✓	✗ (RADAI)	✗	✓	✓	✓	✓	✓	✗	✗ (PtVAS)	✗	✗	✗ (PtVAS)	✓	✗	✗	✗
CONAART (Marcos et al., 2010) (Hiligsmann et al., 2018)*	✓	✓	✓	✗ (RAPID3)*	✓	✗	✓	✓	✓	✗ (RAQoL & RAPID3)*	✗ (PtVAS & RAPID3)*	✓	✗	✗ (PtVAS)	✓	✗	✗	✗
CORRONA (Kremer, 2016)	✓	✓	✓	✗	✗	✗	✗	✓	✓	✗	✗ (PtVAS)	✓	✗	✗ (PtVAS)	✓	✗	✗	✓

Please Note: T/SJC = Tender/Swollen Joint Count, A/LJC = Active/Limited Joint Count, DAS28 = Disease Activity Score-28 Joint count, JADAS = Juvenile Arthritis Disease Activity Score, C/SDAI = Clinical/Simplified Disease Activity Index, RAID = Rheumatoid Arthritis Impact of Disease, (C)HAQ = (Child) Health Assessment Questionnaire , MSK-HQ = Musculoskeletal Health Questionnaire, VAS = Visual Analogue Scale, EQ-5D = EuroQol 5 Dimensions, BRAF = Bristol Rheumatoid Arthritis Fatigue, WSAS = Work and Social Adjustment Scale, RADS = Rheumatoid Arthritis Distress Scale, BRASS = Brigham and Women's Hospital Rheumatoid Arthritis Sequential Study, BSR-BR = British Society of Rheumatology Biologics Register, CAPS = Childhood Arthritis Prospective Study, CARRA = Childhood Arthritis and Rheumatology Research Alliance, CATCH = Canadian Early Arthritis Cohort, CONAART = Argentine Consortium for Early Arthritis, CORRONA = Consortium of Rheumatology Researchers of North America, PtVAS = Patient Global VAS, SF-12/36 = Short Form – 12/36 Item, ASES = Arthritis Self-Efficacy Scale, MHI = Mental Health Inventory, PHQ-9 = Patient Health Questionnaire 9-items, B-IPQ = Brief Illness Perceptions Questionnaire, WPS = Work Productivity Survey, MFQ = Mood and Feelings Questionnaire, PGH-7 = Pediatric Global Health 7-item, RADAI = Rheumatoid Arthritis Disease Activity Index, RAPID3 = Routine Assessment of Patient Index Data 3, RAQoL = RA Quality of Life, *Collected but data Not currently published

Part Three																		
Name and Reference of Registry or Cohort	Treatment History	Clinical Notes	DAS28/JADAS or C/SDAI	RAID	Manifestations/ Features	Steroid Use	Stiffness	T/SJC or A/LJC	Imaging (Radiographs)	MSK-HQ	Pain VAS	EQ-5D	BRAF	Fatigue VAS	(C)HAQ	WSAS	RADS	Treatment Change Reasons
DANBIO (Ibfelt et al., 2016)	✓	✓	✓	×	×	✓	×	✓	✓	×	✓	✓	×	✓	✓	×	×	✓
DREAM (de Punder et al., 2012) (Hiligsmann et al., 2018)*	✓	✓	✓	×	×	✓	×	✓	×	×	× (PtVAS)	×	×	× (PtVAS)	✓	×	× (SF-6D)*	×
ERAS and ERAN, (Young et al., 2011)	Mainly csDMARD	✓	✓	× (SF-36)	✓	✓	×	✓	✓	× (SF-36)	× (PtVAS)	× (SF-36)	× (SF-36)	× (SF-36)	✓	× (SF-36)	× (SF-36)	✓
ESPOIR (Combe et al., 2007)	✓	✓	✓	× (AIMS2)	×	×	×	✓	✓	× (AIMS2)	× (PtVAS & AIMS2)	✓	× (SF-36)	× (SF-36)	✓	× (AIMS2 & SF-36)	× (SF-36)	×
GISEA (Lapadula et al., 2011)	✓	✓	✓	×	✓	✓	×	✓	✓	×	× (PtVAS)	✓	×	× (PtVAS)	✓	× (work time lost)	×	✓
GLADAR (Massardo et al., 2012)	×	×	✓	×	×	×	×	✓	✓	×	✓	×	×	× (PtVAS)	✓	×	×	×
ICON (Sengler et al., 2015)	✓	✓	✓	× (PedsQL)	✓	✓	✓	✓	×	× (PedsQL)	✓	× (PedsQL)	×	× (PtVAS)	✓	×	×	×
IMPARTS (Matcham et al., 2016; Rayner et al., 2014)	×	✓	✓	× (SF-36)	×	×	×	✓	×	× (SF-36)	✓	×	× (SF-36)	✓	✓	× (SF-36)	× (PHQ9, GAD7 & SF-36)	×

Please Note: T/SJC = Tender/Swollen Joint Count, A/LJC = Active/Limited Joint Count, DAS28 = Disease Activity Score-28 Joint count, JADAS = Juvenile Arthritis Disease Activity Score, C/SDAI = Clinical/Simplified Disease Activity Index, RAID = Rheumatoid Arthritis Impact of Disease, (C)HAQ = (Child) Health Assessment Questionnaire , MSK-HQ = Musculoskeletal Health Questionnaire, VAS = Visual Analogue Scale, EQ-5D = EuroQol 5 Dimensions, BRAF = Bristol Rheumatoid Arthritis Fatigue, WSAS = Work and Social Adjustment Scale, RADS = Rheumatoid Arthritis Distress Scale, DANBIO = Danish biologics register for rheumatologic diseases, DREAM = Dutch Rheumatoid Arthritis Monitoring registry, ERAS/N = Early Rheumatoid Arthritis Study/Network, ESPOIR = Etude et Suivi des Polyarthrites Indifférenciées Récentes, GISEA = Italian Group for the Study of Early Arthritis, GLADAR = Grupo Latino Americano de Estudio de Artritis Reumatoide, ICON = Inception Cohort of Newly diagnosed patients with JIA, IMPARTS = Integrating Mental & Physical healthcare: Research, Training & Services, PtVAS = Patient Global VAS, SF-6D = Short Form – 6 Dimensions, SF-36 = Short Form – 36 Item. AIMS2 = Arthritis Impact Measurement Scales 2, Peds-QL = Pediatric Quality of Life Inventory, PHQ9 = Patient Health Questionnaire 9-item, GAD7 = Generalised Anxiety Disorder 7-item, *Collected but data Not currently published

Name and Reference of Registry or Cohort	Treatment History	Clinical Notes	DAS28/JADAS or C/SDAI	RAID	Manifestations/ Features	Steroid Use	Stiffness	T/SJC or A/LJC	Imaging (Radiographs)	MSK-HQ	Pain VAS	EQ-5D	BRAF	Fatigue VAS	(C)HAQ	WSAS	RADS	Treatment Change Reasons
									Part Three									
IORRA (Yamanaka et al., 2007) (Hiligsmann et al., 2018)*	✓	✓	✓	×	×	✓	×	✓	×	×	✓	✓	×	×	✓	×	×	×
JACS (Beukelman, Anink, et al., 2017)	Not Clear	Not Clear	✓	×	✓	Not Clear	Not Clear	✓	×	×	✓	×	×	× (PtVAS)	✓	×	×	Not Clear
JUMBO (Minden et al., 2012)	✓	✓	× (PhVAS)	×	✓	✓	✓	✓	×	× (PtVAS)	✓	×	× (PtVAS)	✓	✓	× (PtVAS)	× (PtVAS)	✓
METEOR (van den Berg et al., 2014)	✓	✓	✓	× (RAP ID3)	×	✓	×	✓	×	× (RAPID 3)	✓	×	×	× (PtVAS)	✓	×	×	✓
NDB (Zink et al., 2001) (Hiligsmann et al., 2018)*	✓	✓	✓	✓	×	✓	×	✓	×	×	✓	✓	×	× (PtVAS)	✓	× (study specific)	×	×
NDB (Wolfe & Michaud, 2011)	✓	✓	× (RA Activity & RADAI)	×	×	✓	✓	✓	×	× (PtVAS)	✓	✓	× (PtVAS)	✓	✓	× (PtVAS)	× (PtVAS)	✓
NEIAA (British Society of Rheumatology, 2019)	Some	✓	✓	×	×	✓	× (MSK -HQ)	✓	×	✓	× (MSK-HQ)	×	×	× (MSK-HQ)	✓	× (WPAI)	× (PHQ4 ADS)	×

Please Note: ESR = Erythrocyte Sedimentation Rate, CRP = C-Reactive Protein, T/SJC = Tender/Swollen Joint Count, A/LJC = Active/Limited Joint Count, DAS28 = Disease Activity Score-28 Joint count, JADAS = Juvenile Arthritis Disease Activity Score, C/SDAI = Clinical/Simplified Disease Activity Index, RAID = Rheumatoid Arthritis Impact of Disease, (C)HAQ = (Child) Health Assessment Questionnaire , MSK-HQ = Musculoskeletal Health Questionnaire, VAS = Visual Analogue Scale, EQ-5D = EuroQol 5 Dimensions, BRAF = Bristol Rheumatoid Arthritis Fatigue, WSAS = Work and Social Adjustment Scale, RADS = Rheumatoid Arthritis Distress Scale, IORRA = Institute of Rheumatology, Rheumatoid Arthritis, JACS = Juvenile arthritis cohort study, JUMBO = Juvenile arthritis MTX/Biologics long-term Observation, METEOR = Measurement of Efficacy of Treatment in the “Era of Outcome” in Rheumatology, NDB = national database of the German Collaborative Arthritis Centres, NDB = National Data Bank for Rheumatic Diseases, NEIAA = National Early Inflammatory Arthritis Audit, PtVAS= Patient Global VAS, PhVAS = Physician Global VAS, RAPID3 = Routine Assessment of Patient Index Data 3, RADAI = Rheumatoid Arthritis Disease Activity Index, WPAI = Work Productivity and Activity Impairment, PHQ4ADS = Patient Health Questionnaire 4 item Anxiety and Depression Screener, *Collected but data Not currently published

Name and Reference of Registry or Cohort	Part Three<br>Treatment History	Clinical Notes	DAS28/JADAS or C/SDAI	RAID	Manifestations/ Features	Steroid Use	Stiffness	T/SJC or A/LJC	Imaging (Radiographs)	MSK-HQ	Pain VAS	EQ-5D	BRAF	Fatigue VAS	(C)HAQ	WSAS	RADS	Treatment Change Reasons
Nijmegen Early RA Cohort (Welsing & van Riel, 2004) (Hiligsmann et al., 2018)*	✓	✓	✓	✖ (AIMS)	✖	✖	✖	✓	✓	✖ (AIMS)	✓	✓	✖ (SF-36)*	✖ (PtVAS)	✓	✖ (AIMS & SF-36)*	✖ (SF-36)*	✖
NoAR (Druce et al., 2015; Symmons & Silman, 2003)	Mainly csDMARD	✓	✓	✖	✖	✓	✖	✓	✓	✖	✓	✖	✖ (SF-36)	✓	✓	✖ (SF-36)	✖ (SF-36)	✖
Nordic JIA Cohort (Nordal et al., 2011)	✓	✓	✓	✖ (SF-36)	✓	✓	✖	✓	✖	✖ (CHQ)	✓	✖ (SF-36)	✖ (SF-36)	✖ (PtVAS)	✓	✖ (SF-36)	✖ (SF-36)	✖
NoRDMARD (Olsen et al., 2014)	✓	✓	✓	✓	✖	✓	✖	✓	✓	✖	✓	✓	✖	✓	✓	✖ (WPAI)	✖	✖
NPRD (Minden et al., 2002)	Mainly csDMARD	✓	✖ (PhVAS)	✖	✖	✓	✖	✓	✖	✖	✓	✖	✖	✖ (PtVAS)	✓	✖	✖	✖
ORAR (Kvien & Uhlig, 2004)	✓	✓	✓	✖ (AIMS2 & SF-36)	✓	✖	Not Clear	✓	✓	✖ (AIMS2 & ASES)	✓	✖	✖ (SF-36)	✓	✓	✖ (AIMS2 & SF-36)	✖ (RAI, AIMS2 & SF-36)	✖
PHARMACHILD (Beukelman, Anink, et al., 2017)	Not Clear	Not Clear	✓	✖	✓	Not clear	Not Clear	✓	✓	✖	✓	✖	✖	✖ (PtVAS)	✖	✖	✖	Not clear

Please Note: T/SJC = Tender/Swollen Joint Count, A/LJC = Active/Limited Joint Count, DAS28 = Disease Activity Score-28 Joint count, JADAS = Juvenile Arthritis Disease Activity Score, C/SDAI = Clinical/Simplified Disease Activity Index, RAID = Rheumatoid Arthritis Impact of Disease, (C)HAQ = (Child) Health Assessment Questionnaire , MSK-HQ = Musculoskeletal Health Questionnaire, VAS = Visual Analogue Scale, EQ-5D = EuroQol 5 Dimensions, BRAF = Bristol Rheumatoid Arthritis Fatigue, WSAS = Work and Social Adjustment Scale, RADS = Rheumatoid Arthritis Distress Scale, NoAR = Norfolk Arthritis Register, NoRDDMARD = Norwegian Antirheumatic Drug Register, NPRD = National Paediatric Rheumatology Database, ORAR = Oslo Rheumatoid Arthritis Register, PHARMACHILD = Pharmacovigilance in JIA patients treated with biologic agents and/or MTX, AIMS (2) = Arthritis Impact Measurement Scales (2), PtVAS = Patient Global VAS, SF-36 = Short Form – 36 Item, CHQ = Child Health Questionnaire, WPAI = Work Productivity and Activity Impairment, PhVAS = Physician Global VAS, RAI = Rheumatology Attitude Index, AIMS2 = Arthritis Impact Measurement Scales 2, SF-36 = Short Form – 36 Item, *Collected but data Not currently published

Part Three

Name and Reference of Registry or Cohort	Treatment History	Clinical Notes	DAS28/JADAS or C/SDAI	RAID	Manifestations/ Features	Steroid Use	Stiffness	T/SJC or A/LJC	Imaging (Radiographs)	MSK-HQ	Pain VAS	EQ-5D	BRAF	Fatigue VAS	(C)HAQ	WSAS	RADS	Treatment Change Reasons
QUEST-RA (Sokka et al., 2007)	✓	✓	✓	×	✓	✓	✓	✓	×	×	✓	×	×	✓	✓	×	×	×
RABBIT (Gerhold et al., 2015)	✓	✓	✓	× (SF-36)	×	×	×	✓	×	× (SF-36)	✓	×	× (SF-36)	✓	× (HFSQ	× (SF-36)	× (SF-36)	×
RADIUS1 (Gibofsky et al., 2011)	✓	✓	✓	×	×	✓	×	✓	×	×	✓	×	×	× (PtVAS)	✓	×	×	✓
ReACCh Out (Oen et al., 2009)	✓	✓	×	× (JAQQ)	✓	✓	×	✓	×	× but JAQQ abd QMLQ	✓	×	×	× (PtVAS)	✓	× (JAQQ)	× (JAQQ)	×
REACH (Alves et al., 2011; Kuijper et al., 2014)	✓	✓	✓	×	×	×	×	✓	×	× (MHLC & SF-36)	× (RADAI)	×	× (FAS)	✓	✓	× (SF-36)	× (SF-36, CORS & HADS)	×
REUMA.PT (Canhão et al., 2011)	✓	✓	✓	×	✓	×	×	✓	✓	× (SF-36)	✓	×	× (SF-36)	×	✓	× (SF-36)	× (SF-36)	✓
Rhumadata© (Choquette et al., 2019)	✓	✓	✓	×	×	✓	✓	✓	×	×	✓	×	×	✓	✓	×	×	✓
ROB-FIN (Nordström et al., 2006)	✓	✓	✓	×	×	✓	×	✓	×	×	✓	×	×	× (PtVAS)	✓	×	×	✓

Please Note: T/SJC = Tender/Swollen Joint Count, A/LJC = Active/Limited Joint Count, DAS28 = Disease Activity Score-28 Joint count, JADAS = Juvenile Arthritis Disease Activity Score, C/SDAI = Clinical/Simplified Disease Activity Index, RAID = Rheumatoid Arthritis Impact of Disease, (C)HAQ = (Child) Health Assessment Questionnaire , MSK-HQ = Musculoskeletal Health Questionnaire, VAS = Visual Analogue Scale, EQ-5D = EuroQol 5 Dimensions, BRAF = Bristol Rheumatoid Arthritis Fatigue, WSAS = Work and Social Adjustment Scale, RADS = Rheumatoid Arthritis Distress Scale, QUEST-RA = Quantitative Patient Questionnaires in Standard Monitoring of Patients with Rheumatoid Arthritis, RABBIT = German register for long-term observation of biologics in RA, RADIUS1 = Rheumatoid Arthritis Disease-Modifying Antirheumatic Drug Intervention and Utilization Study Cohort1, ReACCh Out = Research in Arthritis in Canadian Children emphasizing Outcomes, ROB-FIN = National Register of Biological Treatment in Finland, SF-36 = Short Form – 36 Item, HFSQ = Hannover Functional Status Questionnaire, PtVAS = Patient Global VAS, JAQQ = Juvenile Arthritis Quality of Life Questionnaire, QMLQ = Quality of My Life Questionnaire, MHLC = Multidimensional Health Locus of Control, RADAI = Rheumatoid Arthritis Disease Activity Index, FAS = Fatigue Assessment Scale.

Part Three																		
Name and Reference of Registry or Cohort	Treatment History	Clinical Notes	DAS28/JADAS or C/SDAI	RAID	Manifestations/ Features	Steroid Use	Stiffness	T/SJC or A/LJC	Imaging (Radiographs)	MSK-HQ	Pain VAS	EQ-5D	BRAF	Fatigue VAS	(C)HAQ	WSAS	RADS	Treatment Change Reasons
RRBR (Codreanu et al., 2014)	✓	✓	✓	×	×	×	×	✓	✓	×	× (PtVAS)	✓	×	× (PtVAS)	✓	× (Work Productivity)	×	✓
SCQM, Switzerland, (Uitz et al., 2000)	✓	✓	✓	×	×	×	✓	✓	✓	×	✓	×	×	× (PtVAS)	✓	×	×	×
SRQ (Eriksson et al., 2014) (Hiligsmann et al., 2018)*	✓	✓	✓	×	×	✓	×	✓	×	×	× (PtVAS)	✓	×	× (PtVAS)	✓	× (WAI)*	× (mFABQ)*	✓
STURE (Askling et al., 2006)	✓	✓	✓	×	×	✓	×	✓	×	×	✓	×	×	× (PtVAS)	✓	×	×	Not clear
Swedish JIA Register (Beukelman, Anink, et al., 2017)	Not Clear	Not Clear	✓	No	✓	Not clear	Not Clear	✓	×	×	✓	×	×	× (PtVAS)	✓	×	×	Not clear
TURKBIO (Önen et al., 2022)	✓	Some	✓	×	×	✓	×	✓	✓	×	✓	× (SF-36)	× (SF-36)	× (SF-36)	✓	× (SF-36)	× (SF-36)	✓
VARAR (Mikuls et al., 2010)	✓	✓	✓	× (RAPID3)	✓	✓	×	✓	×	×	✓	×	×	×	✓	×	×	×
WC (Kovalchik et al., 2012)	Mainly csDMARD	✓	✓	× (PSH)	✓	×	✓	✓	✓	× (PSH)	✓	×	×	✓	✓	× (PSH)	× (PSH)	×

Please Note: T/SJC = Tender/Swollen Joint Count, A/LJC = Active/Limited Joint Count, DAS28 = Disease Activity Score-28 Joint count, JADAS = Juvenile Arthritis Disease Activity Score, C/SDAI = Clinical/Simplified Disease Activity Index, RAID = Rheumatoid Arthritis Impact of Disease, (C)HAQ = (Child) Health Assessment Questionnaire , MSK-HQ = Musculoskeletal Health Questionnaire, VAS = Visual Analogue Scale, EQ-5D = EuroQol 5 Dimensions, BRAF = Bristol Rheumatoid Arthritis Fatigue, WSAS = Work and Social Adjustment Scale, RADS = Rheumatoid Arthritis Distress Scale, ROB-FIN = National Register of Biological Treatment in Finland, RRBR = Romanian Registry of Rheumatic Diseases, SCQM = Swiss Clinical Quality Management in Rheumatic Diseases, SRQ = Swedish Rheumatology Quality Registries, STURE = Stockholm TNF follow-Up Registry, TURKBIO = Turkish Biologics Register, VARAR = Veterans Affairs Rheumatoid Arthritis Registry, WC = Western Consortium of Practicing Rheumatologists, PtVAS = Patient Global VAS, WAI = Work Ability Index , mFABQ = modified Fear Avoidance-Belief Questionnaire, SF-36 = Short Form – 36 Item, RAPID3 = Routine Assessment of Patient Index Data 3, PSH = Patient overall satisfaction with health, *Collected but data Not currently publishe
