# Supplementary material for: Refractory inflammatory arthritis definition and model generated through patient and multi-disciplinary professional modified Delphi process
Source: PLoS One. 2023 Aug 9;18(8):e0289760. doi: 10.1371/journal.pone.0289760 (PMC10411820; doi:10.1371/journal.pone.0289760)
Supplement: S2 Data — (PDF) [file pone.0289760.s010.pdf]

Supplementary Figure S3: Example of Delphi Voting Questions (Round One Online)

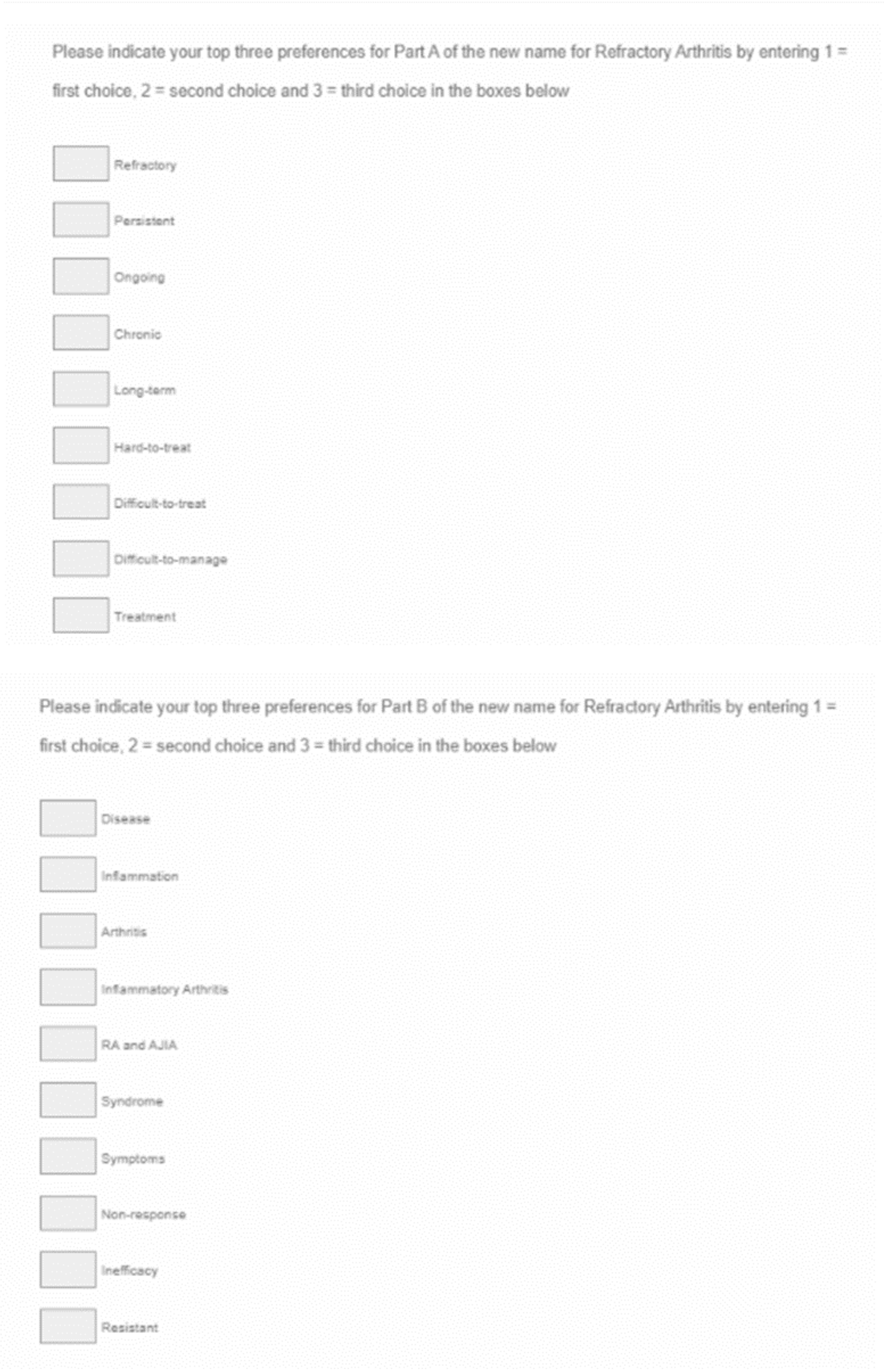

Please indicate your top three preferences for Part A of the new name for Refractory Arthritis by entering 1 = first choice, 2 = second choice and 3 = third choice in the boxes below

- [ ] Refractory
- [ ] Persistent
- [ ] Ongoing
- [ ] Chronic
- [ ] Long-term
- [ ] Hard-to-treat
- [ ] Difficult-to-treat
- [ ] Difficult-to-manage
- [ ] Treatment

Please indicate your top three preferences for Part B of the new name for Refractory Arthritis by entering 1 = first choice, 2 = second choice and 3 = third choice in the boxes below

- [ ] Disease
- [ ] Inflammation
- [ ] Arthritis
- [ ] Inflammatory Arthritis
- [ ] RA and AJIA
- [ ] Syndrome
- [ ] Symptoms
- [ ] Non-response
- [ ] Inefficacy
- [ ] Resistant

Part 1 - Treatment

DESPITE following Treat-to-Target Strategy using treatment with csDMARDs, anti-TNF, bDMARDs and/or tsDMARDs with different mechanisms of action

In your opinion, does the above statement need to be kept broad as it is or have different numbers of drugs for presence (Refractory Inflammation) or absence (Refractory Symptoms) of inflammation?

- ◯ Broad (As it is)
- ◯ Different numbers for Refractory Inflammation and Refractory Symptoms

In your opinion, does the above statement need to be kept broad as it is or have different numbers of drugs for different stages? (e.g. ≥2 csDMARDs and ≥2 b/tsDMARDs (Roodenrijs et al. (2018)) for Stage 1, then increasing for each stage)

- ◯ Broad (As it is)
- ◯ Different numbers to allow for Staging

In your opinion, does the above statement need to be kept broad as it is or have different numbers of drugs for RA versus AJIA? (e.g. ≥2 csDMARDs and ≥3 b/tsDMARDs for RA and ≥1 csDMARDs and ≥3 b/tsDMARDs (Kearsley-Fleet et al. (2018))

- ◯ Broad (As it is)
- ◯ Different numbers for RA and AJIA

Part 2 – Inflammation

PRESENCE (Refractory Inflammation) or ABSENCE (Refractory Symptoms) of synovial inflammation (determined by inflammatory markers, physical examination and imaging or composite disease activity score)

In your opinion, does the above statement need to be kept broad as it is or specify cut-offs or ranges?

- ◯ Broad (As it is)
- ◯ Specify cut-offs or ranges

Domain 1: Pain

Please rate whether **Pain** as a domain should be included as part of Definition of Refractory Arthritis (or newly voted name TBC)?

	Definitely Not Include								Definitely Include
	1	2	3	4	5	6	7	8	9
**Pain**	○	○	○	○	○	○	○	○	○

For each of the Pain components, please rate how related they are for assessing **Refractory Arthritis** (or newly voted name TBC)?

	Highly Unrelated			Neither Related or Unrelated			Highly Related
	-3	-2	-1	0	1	2	3
**Pain during the day**	○	○	○	○	○	○	○
**Pain during the night**	○	○	○	○	○	○	○
**Pain when active**	○	○	○	○	○	○	○
**Pain when resting**	○	○	○	○	○	○	○
**Pain in joints** e.g. hands and feet	○	○	○	○	○	○	○
**Pain in other areas** e.g. muscles, neuropathic, regional (e.g. back), widespread etc	○	○	○	○	○	○	○

For each of the Pain components, please rate how related they are for assessing **Disease Flare**?

	Highly Unrelated			Neither Related or Unrelated			Highly Related
	-3	-2	-1	0	1	2	3
**Pain during the day**	○	○	○	○	○	○	○
**Pain during the night**	○	○	○	○	○	○	○
**Pain when active**	○	○	○	○	○	○	○
**Pain when resting**	○	○	○	○	○	○	○
**Pain in joints** e.g. hands and feet	○	○	○	○	○	○	○
**Pain in other areas** e.g. muscles, neuropathic, regional (e.g. back), widespread etc	○	○	○	○	○	○	○

Does the wording for any of the Pain components need clarifying or amending?

☐ No, the wording for all Pain components is clear and understandable

☐ Yes, please specify which changes are needed to which components
