# Supplementary material for: Refractory inflammatory arthritis definition and model generated through patient and multi-disciplinary professional modified Delphi process
Source: PLoS One. 2023 Aug 9;18(8):e0289760. doi: 10.1371/journal.pone.0289760 (PMC10411820; doi:10.1371/journal.pone.0289760)
Supplement: S3 Data — (PDF) [file pone.0289760.s011.pdf]

Supplementary Table S5: Options for Name preference voting with Proportions and 95% Confidence Intervals

Nominal Group (n=12)†		Online Round 1 (n=40)				Online Round 2 (n=53)	
Name	Proportion of Votes [95%CIs]	Part A Name	Proportion of Votes [95%CIs]	Part B Name	Proportion of Votes [95%CIs]	Name	Proportion of Votes [95%CIs]
Refractory disease	0.42 [0.20, 0.63]	A1 Refractory	0.26 [0.20, 0.32]	B1 Disease	0.25 [0.19, 0.30]	Refractory Disease	0.14 [0.10, 0.17]
Persistent disease	0.21 [0.03, 0.39]	A2 Persistent	0.23 [0.17, 0.28]	B2 Inflammation	0.07 [0.04, 0.11]	Refractory Arthritis	0.11 [0.08, 0.15]
Difficult to treat	0.04 [-0.06, 0.14]	A3 Ongoing	0.04 [0.01, 0.07]	B3 Arthritis	0.22 [0.17, 0.34]	Refractory Inflammatory Arthritis	0.25 [0.20, 0.29]
Chronic disease	0.04 [-0.06, 0.14]	A4 Chronic	0.06 [0.03, 0.09]	B4 Inflammatory Arthritis	0.19 [0.14, 0.24]	Persistent Disease	0.08 [0.05, 0.11]
Treatment non-responsive	0.04 [-0.06, 0.14]	A5 Long-term	0.02 [0.00, 0.03]	B5 RA and AJIA	0.13 [0.08, 0.17]	Persistent Arthritis	0.08 [0.05, 0.11]
Treatment Inefficacy	0.04 [-0.06, 0.14]	A6 Hard-to-treat	0.08 [0.05, 0.12]	B6 Syndrome	0.02 [0.00, 0.03]	Persistent Inflammatory Arthritis	0.19 [0.15, 0.24]
Treatment failure	0.00 [-0.02, 0.02]	A7 Difficult-to-treat	0.17 [0.12, 0.22]	B7 Symptoms	0.09 [0.05, 0.13]	Difficult-to-treat Disease	0.03 [0.01, 0.06]
Treatment resistant	0.13 [-0.03, 0.28]	A8 Difficult-to-manage	0.12 [0.07, 0.16]	B8 Non-response	0.01 [-0.01, 0.02]	Difficult-to-treat Arthritis	0.04 [0.02, 0.06]
Hard to treat	0.00 [-0.02, 0.02]	A9 Treatment	0.03 [0.00, 0.05]	B9 Inefficacy	0.00 [0.00, 0.00]	Difficult-to-treat Inflammatory Arthritis	0.08 [0.04, 0.11]
Difficult to manage	0.08 [-0.05, 0.21]			B10 Resistant	0.03 [0.01, 0.06]		

†Note: one missing voter due to late arrival
