# Supplementary material for: Refractory inflammatory arthritis definition and model generated through patient and multi-disciplinary professional modified Delphi process
Source: PLoS One. 2023 Aug 9;18(8):e0289760. doi: 10.1371/journal.pone.0289760 (PMC10411820; doi:10.1371/journal.pone.0289760)
Supplement: S4 Data — (PDF) [file pone.0289760.s012.pdf]

Supplementary Data S9: Refractory Inflammatory Arthritis Definition Components Statistics

Domain and Components	Statistics
1 - Disease Activity	Percentage Include: 94.87% [82.7%, 99.3%]<br>Mean Include: 8.28 [7.96, 8.61]
Persistently high inflammation and/or symptoms e.g. with or without fluctuations for at least two consecutive clinical visits over the period of at least six months (Combined components)	Mean Related: 2.56 [2.37, 2.76]<br>Mean diff=0.92, d=0.67<br>I-CVI = 0.95 (Excellent)
Disease Activity not captured by DAS28 (hands, shoulders, wrists, elbows and knees) including involvement of other joints (hips, TMJ, feet), other inflammatory features (vasculitis, uveitis, tendonitis or enthesitis) or non-inflammatory features (muscle weakness or cachexia)	Percentage Include: 76% [61.8%, 86.9%]<br>Mean Include: 7.48 [6.97, 7.99]<br>I-CVI = 0.76 (Excellent)
Presence of Extra-articular Manifestation(s), Complications or associated pathology of Inflammatory Arthritis e.g. Secondary Sjögrens, Nodules, Interstitial Lung Disease, (JIA-associated) Uveitis, Cardiovascular Disease, Anaemia or Chronic leg ulcers (Combined above)	Percentage Include: 62% [47%, 75%]<br>Mean Include: 6.42 [5.66, 7.18]<br>I-CVI = 0.71 (Good)
Repeated need of short course steroid tablets or intra-articular injections, that may or may not control flare and localised swelling	Percentage Include: 70% [55.4%, 82.1%]<br>Mean Include: 6.94 [6.43, 7.45]<br>I-CVI = 0.76 (Excellent)
2 – Joint Involvement	
2a - Joint Stiffness	Percentage Include: 92.3% [79.1%, 98.4%]<br>Mean Include: 8.21 [7.83, 8.58]
Joint stiffness during the day (lasting longer than 30-60 minutes in the morning)	Mean Related: 2.54 [2.31, 2.77]<br>Mean diff=0.18, d=0.18<br>I-CVI = 0.92 (Excellent)
2b - Joint Activity	Percentage Include: 89.47% [75.2%, 97.1%] Mean Include: 8.21 [7.83, 8.59]
One or two persistently active/affected joints despite acceptable control in other joints	Mean Related: 2.00 [1.69, 2.31]<br>Mean diff=1.11, d=0.78<br>I-CVI = 0.68 (Good)
Accrued damage due to inflammation - Joint erosion(s), deformity(ies) or restrictions in range of movement (may or may not be painful)	Mean Related: 1.37 [0.85, 1.88]<br>Mean diff=1.68, d=0.95<br>I-CVI = 0.54 (Fair)
3 - Pain	Percentage Include: 82.1% [66.5%, 92.5%]<br>Mean Include: 7.79 [7.27, 8.32]
Pain in joints e.g. hands and feet	Mean Related: 2.38 [2.13, 2.64]<br>Mean diff=-0.08, d=-0.10<br>I-CVI = 0.87 (Excellent)
Pain during the day and/or night (Combined components)	Mean Related: 2.10 [1.80, 2.41]<br>Mean diff=0.03, d=0.03<br>I-CVI = 0.85 (Excellent)
Pain Interference impacting on quality of life (Originally part of Functioning and Quality of Life)	Mean Related: 2.16 [1.81, 2.52]<br>Mean diff=0.27, d=0.22<br>I-CVI = 0.76 (Excellent)

Domain and Components	Statistics
4 – Fatigue	Percentage Include: 79.5% [63.5%, 90.7%]<br>Mean Include: 7.54 [6.92, 8.16]
Lack of physical energy resulting in difficulties conducting daily activities e.g. washing, dressing	Mean Related: 2.05 [1.70, 2.40]<br>Mean diff=0.18, d=0.16<br>I-CVI = 0.74 (Excellent)
Lack of mental energy leading to difficulties with concentration and memory	Mean Related: 1.82 [1.45, 2.19]<br>Mean diff=0.28, d=0.20<br>I-CVI = 0.63 (Good)
5 - Functioning and Quality of Life	Percentage Include: 78.4% [61.8%, 90.2%]<br>Mean Include: 7.78 [7.30, 8.26]
Problems with self-care e.g. washing/dressing	Mean Related: 2.11 [1.71, 2.51]<br>Mean diff=0.05, d=0.04<br>I-CVI = 0.73 (Good)
Inability to perform desired activities e.g. hobbies, social, salaried/voluntary work	Mean Related: 2.03 [1.62, 2.43]<br>Mean diff=0.00, d=0.00<br>I-CVI = 0.67 (Good)
Poor physical function e.g. lack of strength, dexterity, grip	Mean Related: 2.03 [1.64, 2.42]<br>Mean diff=0.05, d=0.05<br>I-CVI = 0.73 (Good)
Reduced mobility and/or Problems walking, standing or climbing stairs e.g. driving, use of public transport, needing to sit most of the day	Mean Related: 2.00 [1.62, 2.38]<br>Mean diff=0.00, d=0.00<br>I-CVI = 0.73 (Good)
Disease-related distress e.g. psychological distress related to burden of disease including Physical, Emotional, Social, Treatment/Healthcare Distress	Mean Related: 2.08 [1.72, 2.44]<br>Mean diff=0.41, d=0.32<br>I-CVI = 0.70 (Good)
6 – DMARD Experiences	Percentage Include: 73% [55.9%, 86.2%]<br>Mean Include: 7.41 [6.70,8.11]
Primary inefficacy (no response to DMARD at all) and/or Secondary inefficacy (developed 'resistance' to DMARD over time)	Mean Related: 2.27 [1.83, 2.71]<br>Mean diff=1.49, d=0.85<br>I-CVI = 0.78 (Excellent)
Experience of multiple occurrences of inefficacy, intolerability or discontinuation	Mean Related: 2.00 [1.47, 2.53]<br>Mean diff=1.46, d=0.75<br>I-CVI = 0.76 (Excellent)

Please note that domains and some components were rated on whether to be included (1 = 'Definitely Not Include' to 9 = 'Definitely Include') and the rest of the components were rated on relatedness (-3 = 'Highly Unrelated' to 3 = 'Highly Related'). For I-CVI, modified kappas are presented here with the following interpretation: Fair of .40 to .59; Good of .60–.74; and Excellent > .74.

Components considered for Definition but included in Model	Statistics
Joint stiffness when resting	Mean Related: 1.69 [1.34, 2.04]<br>Mean diff=0.05, d=0.04<br>I-CVI = 0.60 (Good)
Joint replacement(s) and/or fusion(s) due to inflammation	Mean Related: 0.79 [0.21, 1.37]<br>Mean diff=1.26, d=0.68<br>I-CVI = 0.37 (Poor)
Pain in other areas e.g. muscles, neuropathic, regional (e.g. back), widespread etc	Mean Related: 0.85 [0.28, 1.42]<br>Mean diff=0.36, d=0.20<br>I-CVI = 0.39 (Poor)
Fatigue lasting several hours or all day	Mean Related: 1.85 [1.42, 2.27]<br>Mean diff=0.05, d=0.04<br>I-CVI = 0.63 (Good)
General Emotional Distress e.g. suspected or diagnosed Depression or Anxiety	Mean Related: 1.65 [1.18, 2.12]<br>Mean diff=0.65, d=0.44<br>I-CVI = 0.52 (Fair)
Low Self-Efficacy/Esteem/Confidence e.g. perceived low ability to manage and cope with symptoms	Mean Related: 1.27 [0.79, 1.75]<br>Mean diff=0.54, d=0.36<br>I-CVI = 0.35 (Poor)
Reduced Social Support Network, Relationship breakdowns/difficulties or restriction of social participation	Mean Related: 1.11 [0.55, 1.66]<br>Mean diff=0.76, d=0.43<br>I-CVI = 0.36 (Poor)
Isolation e.g. quantity of relationships or from unemployment, or Loneliness e.g. perception of disagreement between actual and desired levels of social contact or perceived quality of relationships	Mean Related: 1.03 [0.44, 1.61]<br>Mean diff=0.73, d=0.40<br>I-CVI = 0.37 (Poor)
Involvement of other inflammatory features outside of DAS28 e.g. Uveitis or Vasculitis (Included above in 1 - Disease Activity)	Percentage Include: 60% [45%, 74%]<br>Mean Include: 6.62 [6.00, 7.24]<br>I-CVI = 0.68 (Good)
Poor Quality Sleep due to Inflammatory Arthritis	Percentage Include: 60% [45%, 74%]<br>Mean Include: 6.46 [5.84, 7.08]<br>I-CVI = 0.44 (Fair)
Inability to taper steroid tablets longer term (steroid dependency)	Percentage Include: 58% [43%, 72%]<br>Mean Include: 6.38 [5.79, 6.97]<br>I-CVI = 0.54 (Fair)
